# Long-term effects of integrated cognitive behavioral therapy for chronic pain: A qualitative and quantitative study

**DOI:** 10.1097/MD.0000000000034253

**Published:** 2023-07-07

**Authors:** Kanako Tsubaki, Kayoko Taguchi, Tokiko Yoshida, Rieko Takanashi, Eiji Shimizu

**Affiliations:** a Graduate School of Medicine, Chiba University, Chiba, Japan; b Research Center for Child Mental Development, Chiba University, Chiba, Japan; c Department of Psychology, Teikyo University, Hachioji, Japan.

**Keywords:** CBT, chronic pain, follow-up, qualitative

## Abstract

Cognitive behavioral therapy (CBT) is known to improve chronic pain management. However, past studies revealed only small to moderate benefits in short-term results, and long-term follow-up studies are lacking. This study aimed to follow an integrated CBT program’s effectiveness 1.5 years after its completion. This observational study was the follow-up on the data collected from our CBT sessions conducted under 3 different studies in 2018 to 2019. Seven assessment items (Numerical Rating Scale, Pain Catastrophizing Scale [PCS], Pain Disability Assessment Scale [PDAS], Patient Health Questionnaire-9 items, Generalized Anxiety Disorder 7, European quality of life 5-dimensions 5-level, and Beck Depression Inventory [BDI]) were statistically analyzed. Thematic analysis was conducted in semi structured interviews. PCS (*F* = 6.52, *P* = .003), PDAS (*F* = 5.68, *P* = .01), European quality of life 5-dimensions 5-level (*F* = 3.82, *P* = .03), and BDI (*F* = 4.61, *P* = .01) exhibited significant changes (*P* < .05), confirmed by pairwise *t* test, revealing a moderate to large effect size. From post-treatment to follow-up, all scores showed no significant changes (*P* > .1). In the qualitative study, the analysis revealed 3 subthemes: “Autonomy,” “Understanding of yourself and pain,” and “Acceptance of pain.” Our study suggests that integrated CBT may reduce the scores of PCS, PDAS and BDI, and this effect lasts for at least 1 year. Identified themes support the relevance of mitigative factors in managing chronic pain.

## 1. Introduction

Global reports indicate that chronic pain (i.e., lasting longer than 3 months) affects an estimated 10% to 15% of the population, and this is increasing every year.^[1]^ Furthermore, many cases develop to be intractable and drug-resistant, and they are associated with persistent or recurrent disability; as a result, societal costs remain very high. There is a vast and imminent need for non-pharmacological, interdisciplinary therapy. Cognitive behavioral therapy (CBT) is considered especially important in the treatment of chronic pain.^[2]^

However, the most recent meta-analyses indicated only small to moderate benefits in short-term results after CBT sessions.^[3]^ Regarding the long-term outcome in the meta-analysis, 15 studies with 1674 participants provided data on the effects of CBT on pain at follow-ups at 6 months or more. Longer-term results are not well reported in the literature, and we observed only 6 related studies published after 2020.^[4–9]^ These studies revealed that the improvements caused by CBT were generally maintained well after 1 year. This discrepancy between small benefits in short-term results and a high level of continuance after 1 year may indicate that there are variants that work after CBT sessions. To resolve this, we conducted qualitative research to analyze patients who finished CBT sessions.

Moreover, there are limited qualitative studies on the effects of CBT. There is only 1 published paper upon searching the queries’ chronic pain’, “CBT,” “qualitative study,” and the ’long term’.^[10]^ However, the study focused on the perspectives of professional stakeholders, who were offering a stepped-care approach for patients with chronic pain.

The present study aims to substantiate the long-term results of two of our previous studies^[11,12]^ and to illuminate the change in patients’ perspectives through qualitative research. In the single-arm trial and randomized control trial (RCT), patients who underwent our newly integrated CBT program for chronic pain exhibited statistically significant improvement with catastrophic cognition. The RCT delivered by videoconferencing suggested that the integrated CBT may reduce pain interference but not pain intensity. We hypothesize that previously reported results are maintained or improved along with the patients’ living, whose cognition is positively converted.

## 2. Methods

### 2.1. Study desigṅ

This longitudinal cohort observational study followed up the data performed by our CBT sessions, conducted as 3 different studies: a prospective open-labeled single-arm trial, an RCT, and a trial with treatment as usual participants from the randomized trial who received rescue CBT treatment.

The first study was in-person CBT, and the second and third were video conference-based CBT. In these sessions, attention-shift training, memory work based on the peak-end rule, sharpening behavioral image training, and video feedback were operated in addition to Conventional CBT programs for chronic pain (including psychoeducation for pain, case formulation for understanding cognitive behavioral models of chronic pain, relaxation exercises such as breathing, and cognitive reconstruction).^[11,12]^ The final CBT sessions for each study finished in 2018, 2019, and 2019, respectively. A total of 41 patients completed the sessions, and they were assessed to be eligible for this study.

### 2.2. Participants and recruitment

Patients were recruited by postal announcements and telephone calls. Participants received packages inside which there were psychological tests and explanation of the study. Next, they were called to confirm their consent, and if they agreed, they joined the study. Then, patients answered psychological tests and sent them back to us. At the same time, they were interviewed by phone, with the duration of about 30 minutes.

Interviews were recorded, and thematic analyses were performed using MAXQDA.

### 2.3. Ethics and dissemination

This study was conducted under the approval of the Research Review Ethic Committee of Chiba University (no. 3899).

Those who wished to participate in the study were informed of its purpose in the postal announcement confirming their willingness. Each patient was notified that participation was voluntary and complete anonymity was provided. They were asked to provide written informed consent. Adverse events included any unfavorable and unintended sign, symptom, or disease temporarily associated with this study, regardless of its relation. All adverse events were reported, and serious adverse events were immediately reported to the Institutional Review Board of Chiba University Hospital and registered with the hospital risk management system.

### 2.4. Outcome measures

Seven assessment items (Numerical Rating Scale [NRS], Pain Catastrophizing Scale [PCS], Pain Disability Assessment Scale [PDAS], Patient Health Questionnaire-9 items [PHQ-9], Generalized Anxiety Disorder 7 [GAD-7], European quality of life 5-dimensions 5-level [EQ-5D-5L], and Beck Depression Inventory [BDI]) were set based on the standard in “Assessment of Chronic Pain” recommended by the International Pain Society.^[13]^

NRS is a self-rated questionnaire that measures pain intensity on a scale of 0 to 10, where 0 = nothing and 10 = severe. For NRS, patients were asked to keep a daily pain diary.

Catastrophizing one’s perception of pain was measured using the PCS. The degree of life disability due to pain was measured using PDAS. Depressive symptoms were assessed with PHQ-9 and BDI-II. GAD-7 is for screening and measuring the severity of generalized anxiety disorder. EQ-5D-5L is a widely applied, valid, and reliable measure of quality of life.

### 2.5. Statistical analysis

Patients were evaluated at week 1 (baseline, Pre-session), week 16 (Post-session), and a follow-up 1-year post-session (FU). For the primary analysis of comparing treatment effects, the means of the least squares and their 95% CIs were estimated by ANCOVA, with the change in the NRS baseline score. ANCOVA model accounted for the variation caused by gender and baseline scores of NRS. For statistical analyses of FU results, a total of 22 patients were compared pre-intervention versus follow-up-intervention. All analyses were performed using JMP 15 PRO, and a 2-tailed alpha level of 0.05 was used to define statistical significance. To assess the long-term effectiveness of CBT on pain, mean total scores at 3 assessment points for all outcomes were analyzed with single-factor (time) repeated measures analysis of variance (ANOVA). Then, 95% CIs were estimated and tested for significant differences by *t* test. Cohen’s d of the change for each variable was collected. All *P* values were 2-sided, and the significance level was set to .05. Pairwise *t* tests were conducted post hoc.

### 2.6. Semi structured interview

In the first part of the interview, participants were asked about their understanding and benefits of the treatment. The second part explored changes in patients. In the third part, patients were asked about their overall impressions of the treatment.

All interviews were conducted by a phone call by the first author (K.T.), a registered psychiatrist. Patients were aware of the professional background of the interviewer. Interviews were audio-recorded.

### 2.7. Qualitative analysis

Thematic analysis^[14]^ was conducted using MAXQDA to manage and organize the data.

In addition to the priori codes and the semi structured interview, 9 codes were identified by an inductive approach. Each recording was listened to several times to establish familiarity. Quotations were extracted to illustrate subthemes. Both positive and negative examples of (sub)themes were coded for and integrated into the analysis during this process. Validity was established through the constant comparative method whereby recordings were repeatedly reviewed to ensure that (sub)themes correspond to the data. The first author undertook this process.

Consolidated criteria for reporting qualitative research were used to double-check the research team’s essential aspects, study methods, study context, findings, analysis, and interpretations.^[15]^

## 3. Results

The sample included 6 men (27%) and 16 women (73%), with a mean age of 50.91 years old (SD = 13.57 years). Participants had chronic pain (median = 14.09 years; SD = 2.11 years) of longstanding duration (Table 1).

**Table 1:** Characteristics of patients.

Patient	In-person/videoconference CBT	Age	Sex	Education history	Family	Site of pain	Duration disease	Mental comorbidity
①	I	78	F	16	5	Legs	28	Depression
②	I	41	M	12	2	Neck, lower back	3	
③	I	34	F	16	3	Legs, chest	16	Anxiety disorder
④	I	44	F	16	3	Legs	10	Depression, eating disorder
⑤	I	79	F	12	1	Lower back, legs	16	
⑥	I	41	M	16	0	Head	2	
⑦	I	60	M	16	2	Arms, shoulder, back	7	
⑧	V	44	F	12	4	Back, neck		
⑨	V	41	M	12	1	Back, head, legs	5	
⑩	V	74	F	12	1	Back, neck, arms	9	
⑪	V	60	F	12	1	Foot	2	
⑫	V	43	F	14	1	Lower back	1	
⑬	V	26	F	16	0	Right side of the body	0.5	
⑭	V	57	F	12	3	Back, mouth	3	Depression, anxiety disorder
⑮	V	62	F	16	0	Back, neck, legs, arms	40	
⑯	V	57	F	16	2	Legs, elbow	13	
⑰	V	51	M	11	3	Arm	3	
⑱	V	45	F	16	1	Lower back	7	
⑲	V	45	F	15	2	Entire body	11	
⑳	V	53	F	18	1	Lower back, back	15	Social anxiety disorder
㉑	V	48	M	12	2	Legs, back	13	Bipolar disorder
㉒	V	37	F	12	1	Lower back	3	Panic disorder

### 3.1. Quantitative study

**Figure 1.**
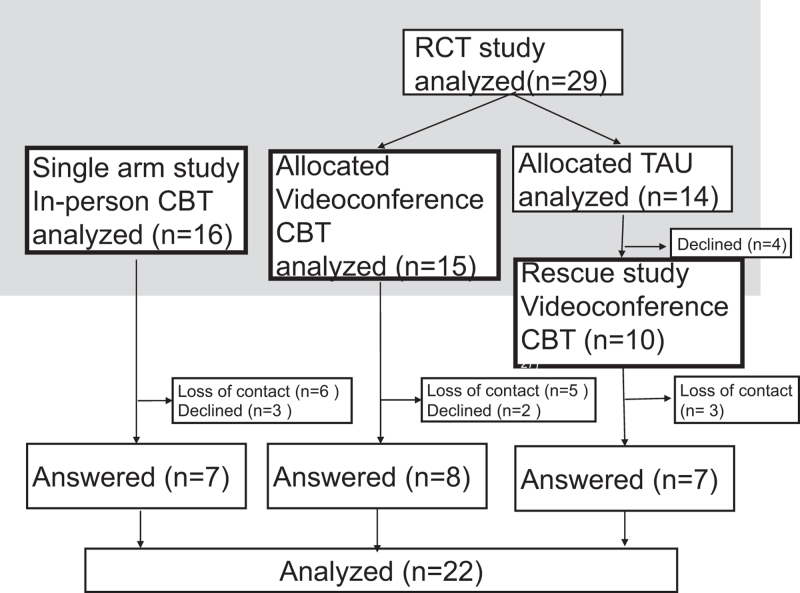
Flow-chart of patient selection. Flow-diagram of the progress from former studies to the current study and final analysis. The number of patients at each step is indicated in parentheses. CBT = cognitive behavioral therapy, RCT = randomized control trial, TAU = treatment as usual.

Figure 1 depicts the patient recruitment for this study; 41 patients previously underwent our CBT intervention program and were assessed to be eligible. However, 14 patients had not responded to the proposition of this research, and 5 patients had declined to participate. In total, 22 patients responded and were interviewed, and FU interview data were analyzed. There were no serious adverse events reported during the study.

Two of the patients lacked NRS scores, and patients 1 to 7 did not answer the score for BDI. Nevertheless, each score at each point is assessed based on the average, meaning that statistical results are not markedly affected.

ANOVA results regarding the long-term effectiveness of CBT showed significant improvement in participant symptoms. Changes in total mean scores from pretreatment to follow-up assessment were –0.82 for NRS (*F* = 1.20, *P* = .031), –8.00 PCS (*F* = 6.52, *P* = .003), –7.59 for PDAS (*F* = 5.68, *P* = .01), –0.77 for PHQ-9 (*F* = 1.17, *P* = .32), −0.73 for GAD-7 (*F* = 2.52, *P* = .09), 0.06 for EQ-5D-5L (*F* = 3.82, *P* = .03), and −4.93 for BDI (*F* = 4.61, *P* = .01). PCS, PDAS, and BDI exhibited significant changes with a moderate to large effect sizes. Next, where repeated measures ANOVA indicated significant changes, pairwise *t* tests were conducted post hoc (Table 2). The results confirmed that a significant change occurred between Pre-CBT and Post-CBT, and there were no significant changes between Post-CBT to FU; this indicates that the effectiveness of CBT was ongoing. Accuracy was tested using the Bonferroni method (*P* < .025).

**Table 2:** Change in psychological scales before, after, and 1 year after CBT sessions.

Variables	Pre		Post		FU		Change from pre to FU					Change from post to FU				
	N	Mean (SD)	N	Mean (SD)	N	Mean (SD)	N	Mean (SD)	95% CI	*P* value	Cohen’s d	N	Mean (SD)	95% CI	*P* value	Cohen’s d
NRS	22	5.03 (1.42)	22	4.46 (1.95)	19	4.18 (2.16)	19	−0.82 (1.69)	–	–	–	19	−0.47 (1.93)	–	–	–
PCS	22	28.22 (11.09)	22	19.77 (9.26)	22	20.23 (8.53)	22	−8.00 (11.33)	4.92	.00	−0.81	22	0.45 (9.94)	3.78	.84	0.05
PDAS	22	21.95 (10.26)	22	14.14 (7.43)	22	14.36 (10.28)	22	−7.59 (10.40)	–	–	–	22	0.23 (7.97)	–	–	–
PHQ-9	22	8.32 (5.09)	22	6.32 (4.28)	22	7.55 (5.75)	22	−0.77 (6.69)	2.26	.60	−0.14	22	1.23 (4.48)	2.55	.22	0.24
GAD-7	22	5.91 (3.89)	22	3.73 (3.02)	22	5.18 (3.49)	22	−0.73 (4.48)	–	–	–	22	1.45 (3.96)	–	–	–
EQ-5D	22	0.60 (0.07)	22	0.66 (0.09)	22	0.65 (0.10)	22	0.06 (0.09)	0.03	.01	0.74	22	−0.01 (0.10)	0.04	.79	−0.06
BDI	15	15.5 (7.44)	15	8.07 (5.71)	15	10.53 (7.89)	15	−4.93 (8.96)	4.12	.06	−0.64	15	2.47 (6.47)	4.36	.18	0.36

BDI = Beck Depression Inventory, CBT = cognitive behavioral therapy, EQ-5D-5L = EuroQol 5 dimensions 5-level, FU = 1 year after CBT, GAD7 = Generalized Anxiety Disorder 7, NRS = numerical range scale, PCS = Pain Catastrophizing Scale, PDAS = Pain Disability Assessment Scale, PHQ-9 = Patient Health Questionnaire-9 items, Post = after CBT, Pre = before CBT.

### 3.2. Qualitative study

**Figure 2.**
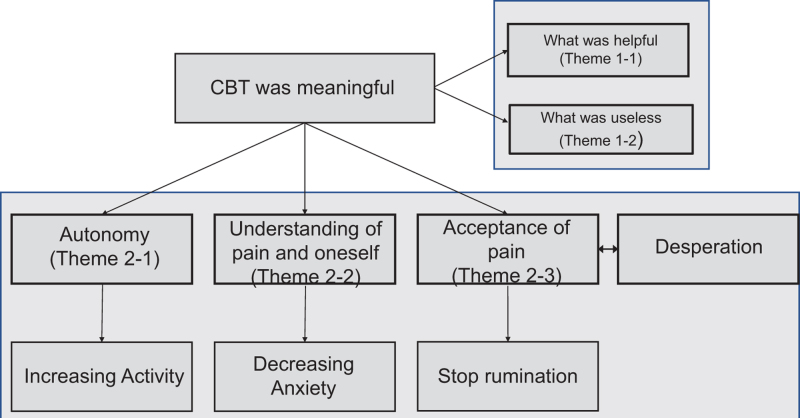
Summary of themes, subthemes, and their inter-relationships. CBT = cognitive behavioral therapy.

The interviews ranged from 10 to 35 minutes. We identified 2 themes and 5 subthemes (outlined in Fig. 2), which are strongly interrelated with nature.

Theme 1: Evaluation of contents

This theme reflects patients’ subjective evaluation of the contents of CBT (16 sessions). Notably, though conceiving that their pain had not been mitigated, all patients evaluated CBT as a helpful treatment; moreover, they were likely to recommend it to other patients.

Theme 1-1: Helpful Contents

Opinions varied in the evaluation, of which contents were helpful or acquired as routine. The most popular ranges on the CBT menu were not indicated. In contrast, many participants emphasized that there was a time during each session when they felt it was counseling and not treatment, which brought a sense of ease.

Many participants described the positive contribution of interactions with therapists.

Thema 1-2: Useless Contents

Participants reported a few contents that were hard to incorporate into daily life, even though they understood the benefits. Meditation and mindfulness are challenging to practice when pain is affecting participants. It was sometimes emotionally challenging for participants to evaluate pain or look back on the history of their pain, as this itself caused pain.

Theme 2: Effect of CBT

This theme reflects physical or mental change after CBT practice and gradual transition after 1 to 2 years.

Thema 2-1: Autonomy

CBT sessions inspire patients to have competence in managing their pain. Before and even after CBT, participants barely attribute pain to psychologic only. However, CBT suggested that psychological conditions affected physical discomfort and, therefore, were manageable to a certain extent. Even though participants do not think all the pain is manageable, fostering a sense of autonomy leads to physical activity increase.

Patient 19

“I was only thinking about getting someone to do something or having something like the hospital or medicine have some effect to get the pain intensity down to zero, but then I was taught CBT, and this showed me how to control the pain mentally, so...I had to do it... yeah. “

2-2: Understanding pain and oneself

CBT has an education program explaining how pain functions and is perceived. Participants confidently described psychoeducation as effective in relieving their anxieties and stopping ruminating.

Patient 8

“It became easier now that I understand the nature of pain. I always knew that chronic pain was painful, but I was told that this is what causes it and that it would be better to avoid it this way, so even if I could not avoid it 100%, it has become easier. I guess you would say I now know how to deal with it (the pain).”

Thema 2-3: Acceptance of pain

Acceptance of pain was the most predominantly mentioned component among the participants. They conceived that their pain would not become 0, so they gradually decided that they should coexist with the pain. In contrast, reference to positive emotions towards medication was infrequent—the loss of expectation for the successful effects of medication is likely to be one of the reasons underlying the acceptance of pain. Participants used the terms “gave up” and “abandoned,” but there is a slight difference between the acceptance of pain and desperation. One of the keys might be to lower one’s self-demand and adjust to the possible range. Nevertheless, further research on this is necessary.

Patient 15

“I have come to think that I can live a normal life even if I am in some pain... In terms of taking the pain away, I think that having some pain is ok...I can live with that.....”

Patient 3

“It’s troublesome to be alive; they say there’s no way to fix the pain, and it won’t go away.”

Integrated findings

**Figure 3.**
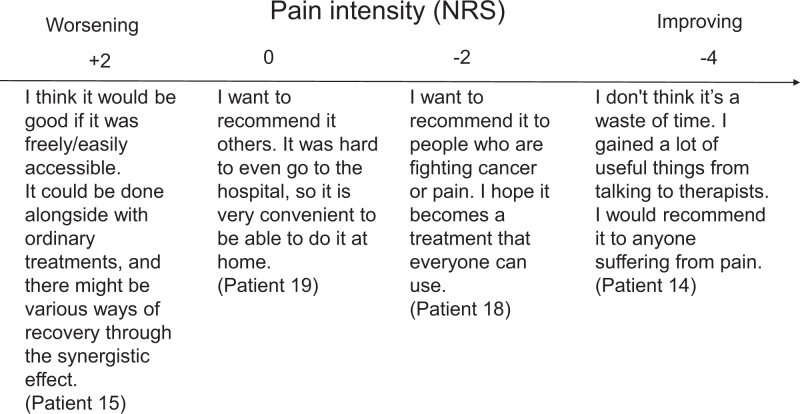
Findings from our mixed study. Citations from patient interviews are included. NRS = Numerical Rating Scale.

All the patients who participated in this study mentioned their satisfaction and expectations for CBT, even though their pain still existed or was even more challenging. However, this may not be applicable to the patients who did not participate (Fig. 3).

## 4. Discussion

This is the first study to combine quantitative and qualitative studies on the long-term effects of CBT on chronic pain. Despite some limitations, the findings of this study indicate that the effects of adjunctive CBT seemed to impact measures of PCS, PDAS, and BDI and are well maintained 1 year after completion of treatment. There are patients who showed a change in their perspectives by gaining autonomy, understanding pain and oneself, and accepting pain. Not all of such patients had experienced mitigation of pain, but these changes in perspective may be the reasons for the decrease in PCS, PDAS and BDI scores.

Firstly, one of the strengths of this study is that we conducted a relatively long-term follow-up. Secondly, our study is an integration of quantitative research and qualitative analysis. Mixed methods research is becoming a vital methodology for complex health-related topics, providing new insights,^[16]^ though currently only a few studies have investigated CBT.^[17–19]^ Thirdly, the interviews were all conducted by telephone, as all participants chose this method over all other communication methods, including the internet (Skype, Zoom, LINE, etc.). This may indicate the relation between internet literacy and the disease rate of chronic pain. If all the interventions of CBT were to be restricted to the internet, then this may result in the potential failure to reach out to participants who need help.

The identified (sub)themes consist of 2 elements: acceptance by education and acquisition of self-efficacy, which both back up former research.^[20]^

Integrated findings show that patients summarized their impressions of therapy as positive, regardless of whether their pain improved or worsened (Fig. 3). There were many patients who claim that CBT is not effective yet maintain high expectations for success in the future and claim CBT to be satisfactory; this disparity is ambiguous and requires further investigation in the future.

This study has several limitations. The main limitations are that most patients who agreed to participate in this study already had a favorable opinion of CBT. There may be patients against CBT but did not answer the proposals of this continued study (14 of the 40 patients that we approached). Therefore, there is a possibility that our results have a selection bias.

Secondly, the sample size is relatively small, and CBT and videoconference-delivered CBT are mixed. The qualitative study had not reached the point of saturation. However, the small sample size is not a weakness of qualitative research but rather a core characteristic of many qualitative methodologies; data are intended to provide rich and deep exploration rather than broad surveys of phenomena. Moreover, *saturation* is an unhelpful, unachievable concept. To replace the idea, there should be a focus on obtaining rich personal accounts and describing the similarities and differences in experiences among participants.^[21]^

Despite the limited effectiveness of CBT, according to meta-analyses, CBT remains one of the only last resorts for ameliorating intractable chronic pain. However, efforts to develop CBT programs for chronic pain and assess their feasibility are remarkably delayed in Asia.^[22]^ Therefore, concurrently with further research, it is necessary to construct systems for disseminating CBT, especially videoconference-delivered CBT.

## Acknowledgments

We express our gratitude and respect for the dedication and contribution of the research participants. We thank our secretary Megumi Murase for her huge support. We thank the English Academic Service at Chiba University for the English editing discussion.

## Author contributions

**Conceptualization:** Kanako Tsubaki, Eiji Shimizu.

**Data curation:** Kanako Tsubaki, Eiji Shimizu.

**Formal analysis:** Kanako Tsubaki, Eiji Shimizu.

**Funding acquisition:** Kanako Tsubaki.

**Investigation:** Kanako Tsubaki, Kayoko Taguchi, Tokiko Yoshida, Rieko Takanashi, Eiji Shimizu.

**Methodology:** Kanako Tsubaki, Eiji Shimizu.

**Project administration:** Kanako Tsubaki, Eiji Shimizu.

**Resources:** Tokiko Yoshida, Rieko Takanashi.

**Supervision:** Eiji Shimizu.

**Validation:** Kanako Tsubaki, Rieko Takanashi, Eiji Shimizu.

**Visualization:** Kanako Tsubaki.

**Writing – original draft:** Kanako Tsubaki.

**Writing – review & editing:** Kanako Tsubaki, Kayoko Taguchi, Tokiko Yoshida, Rieko Takanashi, Eiji Shimizu.
